# The cause of acute lethality of mice exposed to a laser-induced shock wave to the brainstem

**DOI:** 10.1038/s41598-022-13826-6

**Published:** 2022-06-08

**Authors:** Koji Yamamura, Nobuaki Kiriu, Satoshi Tomura, Satoko Kawauchi, Kaoru Murakami, Shunichi Sato, Daizoh Saitoh, Hidetaka Yokoe

**Affiliations:** 1 Department of Oral and Maxillofacial Surgery, National Defense Medical College, Tokorozawa, Japan; 2 Division of Traumatology, Research Institute, National Defense Medical College, Tokorozawa, Japan; 3 Department of Traumatology and Critical Care Medicine, National Defense Medical College, Tokorozawa, Japan; 4 Division of Bioinformation and Therapeutic Systems, Research Institute, National Defense Medical College, Tokorozawa, Saitama Japan

**Keywords:** Medical research, Pathogenesis, Risk factors

## Abstract

Air embolism is generally considered the most common cause of death within 1 h of a blast injury. Shock lung, respiratory arrest, and circulatory failure caused by vagal reflexes contribute to fatal injuries that lead to immediate death; however, informative mechanistic data are insufficient. Here we used a laser-induced shock wave (LISW) to determine the mechanism of acute fatalities associated with blast injuries. We applied the LISW to the forehead, upper neck, and thoracic dorsum of mice and examined their vital signs. Moreover, the LISW method is well suited for creating site-specific damage. Here we show that only mice with upper neck exposure, without damage elsewhere, died more frequently compared with the other injured groups. The peripheral oxygen saturation (SpO_2_) of the former mice significantly decreased for < 1 min [*p* < 0.05] but improved within 3 min. The LISW exposure to the upper neck region was the most lethal factor, affecting the respiratory function. Protecting the upper neck region may reduce fatalities that are related to blast injuries.

## Introduction

Terrorist bombings continue as a major problem worldwide. Although the number of patients with injuries caused by terrorist bombs peaked in 2014, the threat of such injuries continues to threaten military and civilian populations, with ≥ 10,000 bombings each year^1^. Evidence indicates that air embolism is the most common cause of immediate death or death within 1 h of a bombing^2,3^. Further, other fatal injuries that can lead to immediate death include shock lung, respiratory arrest, or circulatory failure caused by vagal reflexes^4–6^; however, not much is known about the underlying mechanisms.

Patients with head trauma or cerebral hemorrhage with complex lesions in the medulla oblongata and other brainstem regions die without ameliorating apnea^7^. Furthermore, in a rat model of severe diffuse brain injury, apnea occurs for approximately 10–20 s immediately after an injury. Respiration subsequently resumes; however, the respiratory rate gradually decreases until death^8,9^.

Our research employs shock and blast tubes to model blast injuries^6,10^. However, it is difficult to produce injuries localized to a specific site, and therefore the cause of immediate death and the responsible organs are unknown. Further, it is difficult to evaluate the response to such injuries inflicted upon each region of the brain.

Here, we used laser-induced shock waves (LISW) to determine the mechanism of acute fatalities caused by blast injuries because the LISW enables site-specific shock wave exposure. We hyphotesized that respiratory failure is caused by injury involving brainstem region; therefore, LISW locally applies shock waves, which enable verification of a site-specific response.

## Methods

### Mice

Male C57BL/6 mice (age: 8–10 weeks, bodyweight: 23–26 g) were obtained from SLC Japan (Shizuoka, Japan). The mice were housed at 22–24 °C with a 12-h light/dark cycle and had free access to food and water. The Animal Ethics Committee of the National Defense Medical College Hospital approved the procedures for using mice (Permission No. 20004). Suffering was minimized by anesthetizing the mice and employing humane endpoints. All experiments were performed in accordance with relevant guidelines and regulations. Our reporting of research involving animals follows the recommendations of the ARRIVE guidelines.

### Experimental procedures

**Figure 1:**
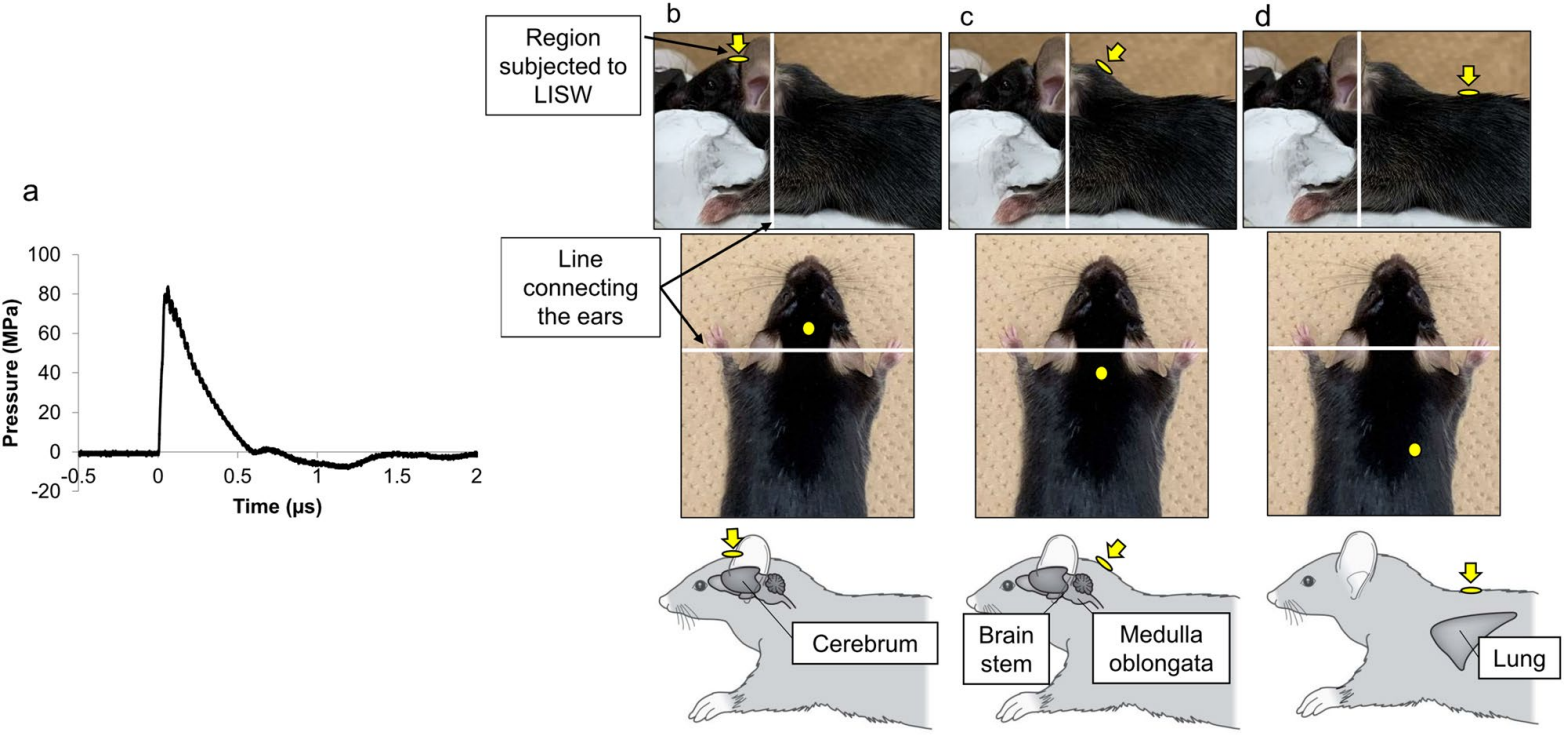
Laser-induced shock wave (LISW) method. (**a**) Typical time–pressure waveform for LISWs with a laser fluence of 3.0 J/cm^2^: Peak pressure, 81.8 MPa; impulse, 19.9 Pa·s; duration, 0.58 μs. (**b**–**d**) Regions subjected to LISWs. (**b**) Frontal region (cerebrum as the target: 5-mm cephalad of the line connecting the ears). (**c**) Upper neck region (brainstem as the target: 5-mm caudal to the line connecting the ears). (**d**) Right thoracic dorsum (right lung as the target: 20-mm caudal to the midline and 5 mm to the right of the the line connecting the ears).

The setup for producing LISW, which is compact and easy to use and control, offers excellent safety and multifunctionality, as well as delivers site-selective shockwave application to laboratory animals at highly reproducible dose^5,11–14^. Furthermore, the positive pressure duration of the LISW used in this study was 0.6 s (Fig. 1), which is much shorter than that of actual explosion shock waves^15^. In this study, we assumed that the impulse of the shock wave, i.e., time-integrated positive pressure component, is a primary parameter to determine brain injury. The impulse of LISW was 20 Pa.s (peak pressure, 82 MPa) (Fig. 1). We applied LISW to the frontal, upper dorsal neck, and right thoracic dorsum (n = 10 per group) (Fig. 1).

### LISW method

1. The experimental mice were anesthetized with 1.5% isoflurane, and the hair at the experimental site, the frontal region, upper dorsal neck, and the right thoracic dorsum was removed with a depilatory cream. Anesthesia was applied until the end of the study or for ≤ 30 min.
2. The mice were placed on a plate in the supine position.
3. A light-absorbing laser target (0.5-mm thick natural black rubber disk) bonded with an optically transparent material (1.0-mm thick polyethylene terephthalate sheet) was placed on the surface of the skin of the frontal, upper dorsal neck, and right thoracic dorsum.
4. A Q-switched ruby laser (The Ruby nano Q, NIIC Co., Tokyo, Japan; 694 nm; pulse-width 20 ns) was used to deliver intense laser pulses, which were used to irradiate the laser target with the required amount of energy.
5. The laser pulse absorbed by the light-absorbing material induced a plasma, whose expansion generated shockwaves.

To establish a mouse model of severe tissue damage, the laser fluence of a LISW was set to 3.0 J/cm^2^.

### Physiological measurements

Heart rate, mean blood pressure (MABP; MK-2000ST; Muromachi, Tokyo, Japan), peripheral oxyhaemoglobin saturation (SpO_2_; Mouse OX; STARR Life Science, Oakmont, PA), respiratory rate (Mouse OX; STARR Life Science, Oakmont, PA) and physiological parameters were noninvasively monitored before and after the application of LISW.

### Pathological assessment

After the application of LISW, the mice were deeply anesthetized with an intramuscular injection of 100 mg/kg ketamine and 10 mg/kg xylazine. The brain was removed after decapitation, fixed in 4% paraformaldehyde for 24 h, and then embedded in paraffin. Sagittal sections (4-μm thick) were cut from the embedded brain and the lung coronal tissues and stained with haematoxylin–eosin (HE). To investigate the cause of lethality in the Upper neck group, Bodian staining was performed on the mice brains of this group as well as those of intact mice for pathological analysis, and the sections were observed using the BZ-X700 light microscope (Keyence Corporation, Osaka, Japan) under 2 × , 10 × and 100 × magnification.

### Statistical analysis

Data are expressed as the mean ± standard deviation (± SD). Vital signs were analyzed using one-way factorial analysis of variance with post-hoc comparisons and Tukey’s test. The survival rates were analyzed using Pearson’s chi-square test. Survival analysis data were compared using the Kaplan–Meier method and log-rank test, and *p* < 0.05 was set to indicate a significant difference. Statistical analysis was performed using Prism 8 (MDF Co., Kameido, Tokyo, Japan).

## Results

### Survival analysis

**Figure 2:**
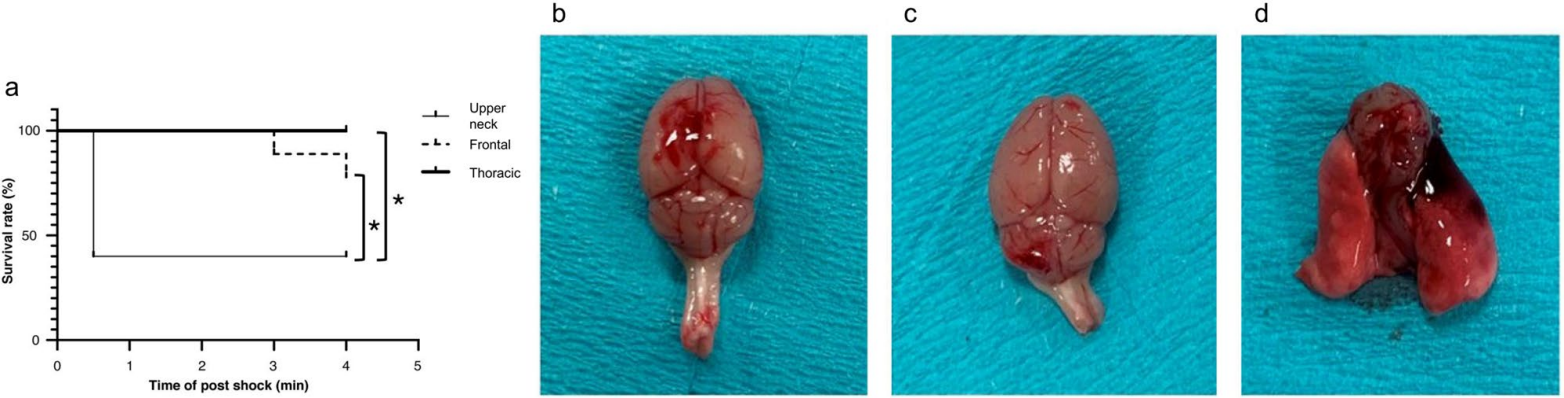
Site-specific characteristics after LISW application. (**a**) Survival of the three groups: Frontal group: All mice died within 3–4 min; Upper neck group: All mice died within 1 min; Thoracic group: No deaths. *p < 0.05 (log-rank test). (**b**, **c**) Extent of macroscopic damage after application of the LISW. (b) Frontal group: Hemorrhage on the surface of the cerebral hemisphere. (**c**) Upper neck group: Hemorrhage on the surface of the cerebellar hemisphere. (**d**) Thoracic group: Hemorrhage in the right lung.

The survival rates of the Frontal, Upper neck, and Thoracic groups, were 80, 40 and 100%, respectively (Table 1). In the Frontal group, all deceased mice experienced respiratory arrest and loss of pulse within 3–4 min. In the Upper neck group, all deceased mice experienced respiratory arrest and loss of pulse within 1 min. With regard to survival time analysis, the upper neck group showed a significant decrease compared to the other two groups (Fig. 2).

**Table 1:** Comparison of post-LISW survival rates.

Number of mice			
Group	Frontal	Upper neck	Thoracic
Survival	8	4	10
Mortality	2	6	0
Total	10	10	10

Survival rates: Frontal group, 80%; Upper neck group, 40%; Thoracic group, 100%.

### Vital signs

Mice for which vital signs were measurable are presented in Table 2. In the group of mice in which LISW was applied to the upper neck region (Upper neck group), the SpO_2_ significantly decrease for < 1 min, increased within 3 min and returned to pre-LISW levels (Table 2). All deceased mice in the Upper neck group developed apnea immediately after LISW application and exhibited marked decreases in SpO2, heart rate, and blood pressure, with no subsequent improvement. In the group of mice in which LISW was applied to the frontal region (Frontal group), the SpO_2_ was unchanged. In the group subjected to LISW to the right-back of the chest region (Thoracic group), the SpO2 decreased to some extent and did not return to pre-LISW values.

**Table 2:** Comparison of post-LISW vital signs.

Minute	Peripheral oxyhemoglobin saturation (%)			Pulse rate (beats per minute)			Mean blood pressure (mmHg)			Respiratory rate (breathes per minute)		
	Frontal (n)	Upper neck (n)	Thoracic (n)	Frontal (n)	Upper neck (n)	Thoracic (n)	Frontal (n)	Upper neck (n)	Thoracic (n)	Frontal (n)	Upper neck (n)	Thoracic (n)
#	97.1 ± 1.4 (10)	97.1 ± 0.8 (10)	96.4 ± 1.4 (10)	389.9 ± 68.7 (10)	413.4 ± 66.1 (10)	355.8 ± 63.6 (10)	91.5 ± 17.2 (10)	97.7 ± 27.6 (10)	91.5 ± 18.5 (10)	110.1 ± 30.3 (10)	112.3 ± 26.4 (10)	141.2 ± 43.7 (10)
0.5	96.6 ± 1.8 (10)	59.9 ± 18.8** (8)	90.1 ± 7.410)	372.2 ± 74.8 (10)	270.7 ± 57.4* (8)	365.7 ± 61.8 (10)				123.3 ± 51.6 (10)	100.6 ± 26.7 (8)	152.1 ± 51.7 (10)
1	96.7 ± 2.7 (10)	75.5 ± 23.7 (4)	92.2 ± 6.1 (10)	374.3 ± 58.8 (10)	347.0 ± 82 (4)	354.5 ± 66.4 (10)	80.7 ± 27.2 (10)	82.5 ± 10.9 (4)	77.5 ± 20.0 (10)	82.8 ± 40.9 (10)	89.0 ± 42.3 (4)	141.3 ± 63.0 (10)
1.5	97.6 ± 0.9 (10)	81.3 ± 27.8 (4)	93.6 ± 4.9 (10)	358.3 ± 65.5 (10)	329.0 ± 67.9 (4)	358.9 ± 73.2 (10)				104.6 ± 62.0 (10)	106.5 ± 60.7 (4)	150.1 ± 52.5 (10)
2	97.5 ± 1.3 (10)	88.6 ± 15 (4)	92.5 ± 6.7 (10)	338.2 ± 78.8 (10)	374.5 ± 119.0 (4)	356.9 ± 76.0 (10)	76.0 ± 21.2 (10)	90.8 ± 47.7 (4)	89.2 ± 36.5 (10)	137.3 ± 88.0 (10)	96.3 ± 67.1 (4)	157.7 ± 58.4 (10)
2.5	96.7 ± 3.7 (10)	85.1 ± 20.1 (4)	92.4 ± 6.4 (10)	341.8 ± 89.9 (10)	344.8 ± 69.9 (4)	352.1 ± 74.1 (10)				106.6 ± 42.9 (10)	139.5 ± 60.4 (4)	114.5 ± 49.5 (10)
3	97.0 ± 2.4 (10)	95.8 ± 2 (4)	91.7 ± 7.5 (10)	337.6 ± 84.1 (10)	335.0 ± 51.7 (4)	362 ± 83.3 (10)	84.4 ± 18.3 (10)	83.0 ± 28.8 (4)	88.5 ± 23.2 (10)	115.2 ± 35.2 (10)	84.5 ± 42.4 (4)	142.8 ± 51.2 (10)
3.5	97.0 ± 1.8 (9)	96.7 ± 0.7 (4)	91.6 ± 7.5 (10)	311 ± 49.6 (9)	357.0 ± 65.0 (4)	354.9 ± 81.3 (10)				110.8 ± 35.3 (9)	148.8 ± 88.4 (4)	117.6 ± 36.6 (10)
4	97.3 ± 1.0 (9)	96.8 ± 0.6 (4)	93.1 ± 5.6 (10)	321.7 ± 60.9 (9)	385.0 ± 119.6 (4)	356.7 ± 77.6 (10)	80.1 ± 23.1 (9)	96.3 ± 7.9 (4)	87.6 ± 13.5 (10)	102.3 ± 34.7 (9)	177.8 ± 133.0 (4)	127.0 ± 44.3 (10)

n: number of animals excluding dead animals in each group. **p < 0.01, vs. Frontal and Thoracic groups. *p < 0.05, vs. Frontal and Thoracic groups. #: Immediately before LISW application.

### Organ injury post-LISW treatment

In mice subjected to 3.0 J/cm^2^ laser fluence, we observed hemorrhage on the surface of the cerebral hemisphere in the Frontal group. A similar hemorrhage was observed on the surface of the cerebellar hemisphere in the Upper neck group, and right-sided lung hemorrhage was observed in the Thoracic group (Fig. 2). No gross bleeding was observed other than at the site of application. There were no fracture lines in the skulls of all injured mice.

### Pathological characteristics

**Figure 3:**
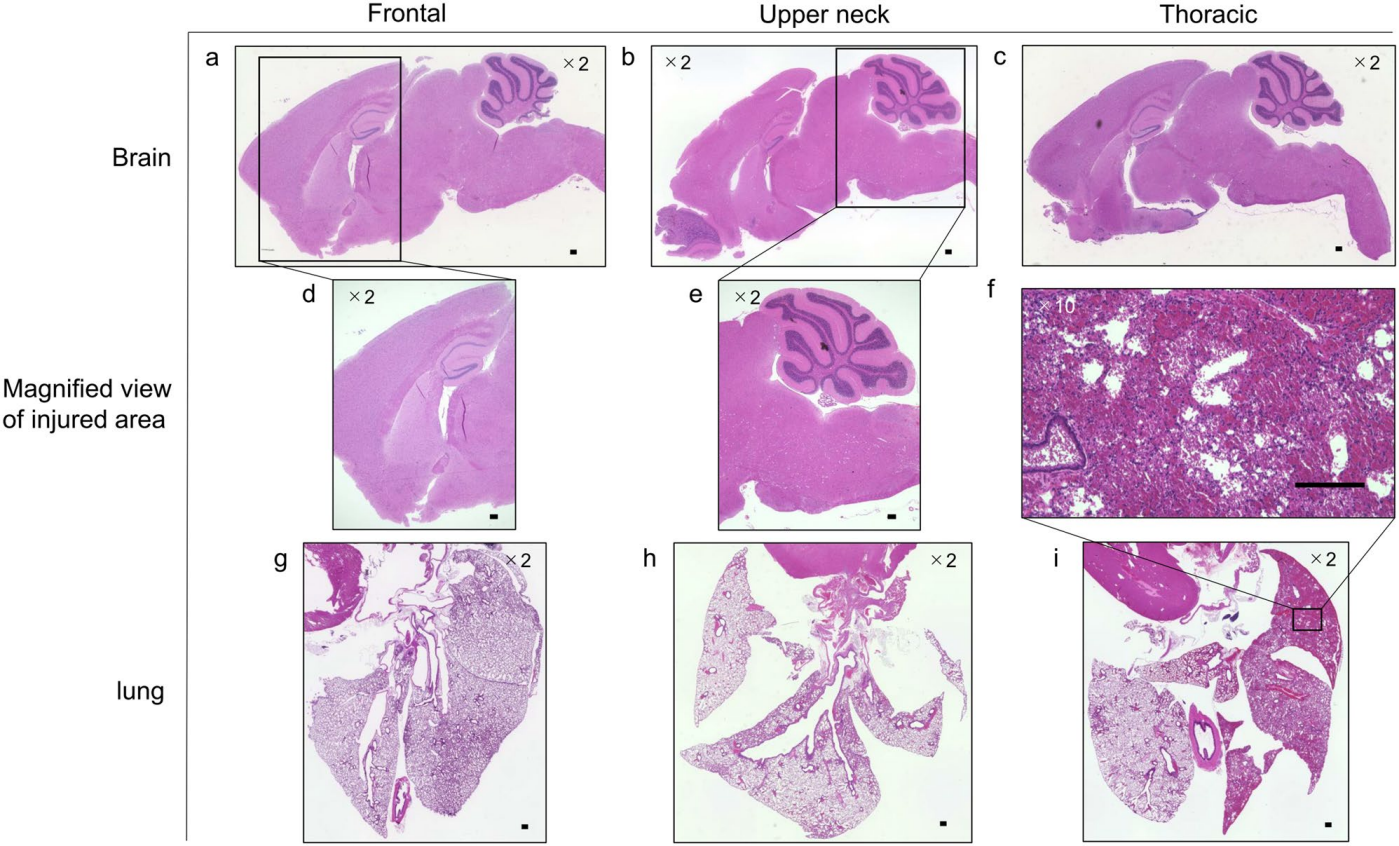
HE-stained brain and lung after application of the LISW. (**a**, **d**) HE-stained brain specimen after application of the LISW in the Frontal group. (**b**, **e**) HE-stained brain specimen after application of the LISW in the Upper neck group. (c) HE-stained brain specimen after application of the LISW in the Thoracic group. (**a**–**e**) No obvious pathological changes were observed. (**f**, **i**) HE-stained lung specimen after application of the LISW in the Thoracic group. There were alveolar hemorrhage associated with alveolar wall elongation and capillary destruction. (**g**) HE-stained lung specimen after application of the LISW in the Frontal group. (**g**) HE-stained lung specimen after application of the LISW in the Upper neck group. (**g**, **h**) No obvious pathological changes were observed. (**a**–**i**) scale 200 μm.

**Figure 4:**
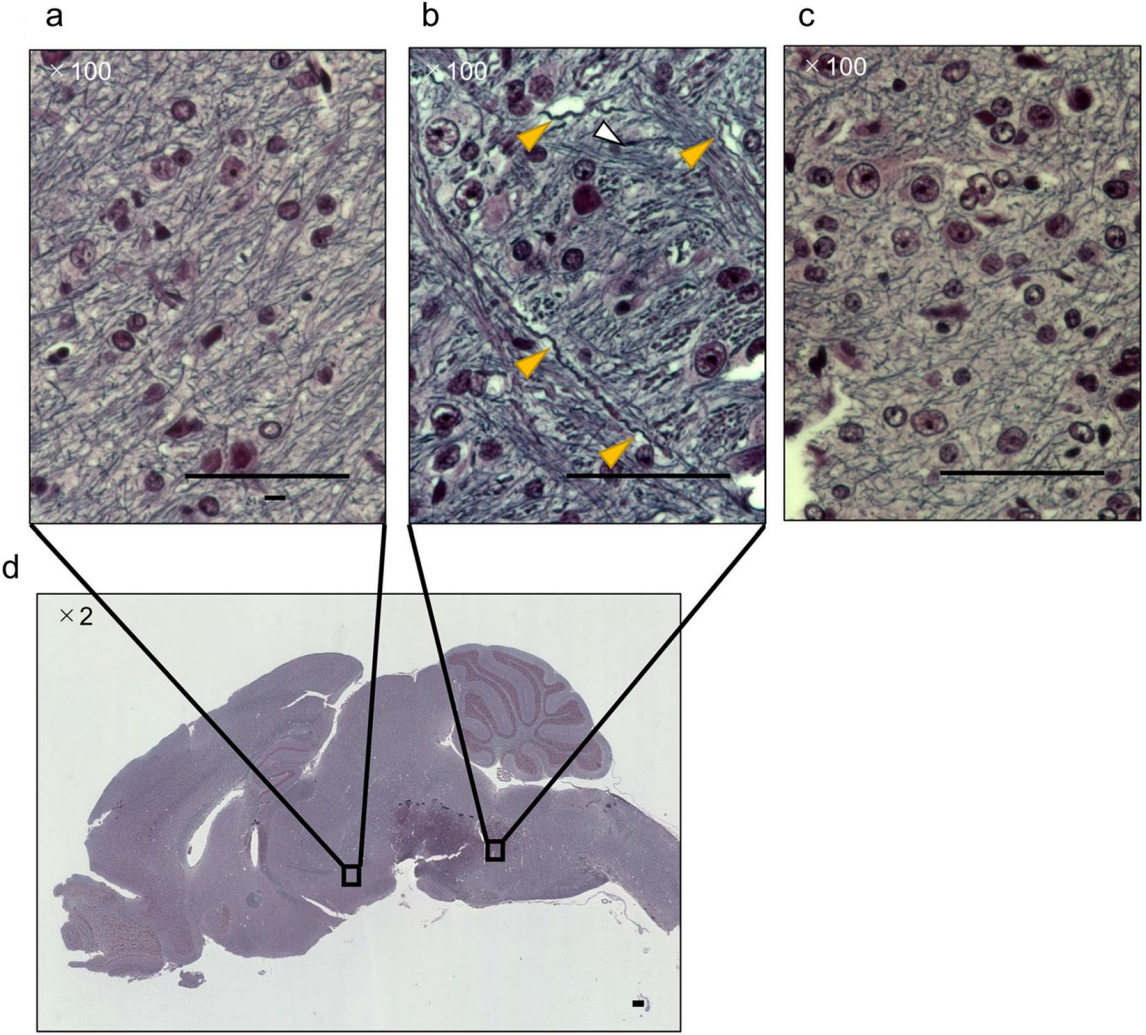
Bodian-stained brain after application of the LISW in the Upper neck group and intact group. (**a**, **b**, **d**) Bodian-stained brain specimen after application of the LISW to the Upper neck group. (**a**) There were no obvious axonal changes in the hypothalamic region. (**b**) Multiple axonal undulations were observed in the medulla oblongata region (yellow arrows). Axonal swellings were observed in several locations (white arrows). (**c**) Bodian-stained brain specimen of an uninjured mouse with no obvious axonal changes in the medulla oblongata region. (**a**–**c**) scale 50 μm. (**d**) scale 200 μm.

No obvious changes were observed in the HE-stained brain specimens after the application of LISW in all the groups as well as at the site of injury, especially in the Frontal and Upper neck groups (Fig. 3). In the Bodian-stained mice brains of the Upper neck group, undulated axons and axonal varicosities, which are normal features of brain injury in the acute phase, were confined to the medulla oblongata (Fig. 4). The Bodian-stained brain specimen of an uninjured mouse exhibited no obvious axonal changes in the medulla oblongata. Regarding the pathological findings of the lungs, the Thoracic group exhibited alveolar hemorrhage associated with alveolar wall elongation and capillary destruction, whereas no obvious changes were noted in the other groups (Fig. 3).

## Discussion

Past evidence indicates that air embolism causes immediate or shortly delayed death in response to a blast injury. Thus, lung damage from an explosion likely causes an embolism^16^. We first expected that a fatal injury would occur when LISW was applied to the lungs, not the head. However, all mice in the Thoracic group survived, although with decreased SpO_2_, in contrast with the mice that survived in the Upper neck group. In addition, the mortality rate was higher in the Upper neck group, suggesting that damage to the upper neck caused respiratory and cardiac arrests. In this study, we similarly demonstrated that respiratory arrest was caused by the disruption of a central nervous mechanism and that injury to this region, including the medulla oblongata, was most likely fatal.

Marmarou et al.^8^ reported that respiratory depression and subsequent hypotension are the main causes of death in a rat model of severe diffuse brain injury and that respiratory depression may be associated with the central vs the peripheral nervous system. Others reported^17^ that direct cranial severe blast injury causes immediate death, which may be explained by primary brainstem failure; however, a neuropathological analysis was not performed. Here we similarly show that respiratory arrest was caused by the disruption of a central nervous mechanism and that the injury to the region, including the medulla oblongata, was most likely fatal. Further, our results suggest that the damage to the upper neck region caused respiratory and cardiac arrest.

Models of severe brain injury that directly impacted the dura mater exhibited significant hypertension immediately after injury^18^. However, models of severe, diffuse brain injury, such as the weight-drop model, caused hypotension immediately after the injury^8^. Our present results demonstrate that blood pressure decreased after the application of the LISW to the head region. Cernak et al.^19^ found that, in a rat model of moderate diffuse brain injury, there was a transient increase in the blood pressure for 5 s after the injury, followed by a significant reduction 1 min after the injury^19^.

Apnea, bradycardia and hypotension occur in thoracic blast injuries, and pulmonary C fibers may be involved^20–22^. Further, hypotension is caused by bradycardia but not by efferent stimulation of the vagal nerve^23,24^, which were not apparent here. Moreover, we expected that the vagal reflex may be induced by unilateral stimulation, such as the application of a right-dorsal LISW. In a rat model of blast-induced traumatic brain injury, bradycardia is observed immediately after injury because of the vagal reflex^25^. Here, we found that the heart rate decreased when LISW was applied to the frontal and upper neck areas. Thus, certain neural reflexes may have caused circulatory changes when the head was exposed to shock waves.

It has been reported that dysfunction associated with brainstem injury may present as abnormal brainstem reflexes, impaired consciousness, respiratory failure, and autonomic failure. Ataxic respiration (irregular pauses and periods of apnea) and central apnea have been observed, particularly in rostral–ventral medullary cord injuries, and they are associated with poor prognoses^26^. Cardiac arrhythmias also occur frequently following brainstem injuries and are associated with increased mortality^27^. A well-known brainstem reflex is the trigeminocardiac reflex (TCR), a unique brainstem reflex that manifests as a sudden decrease in heart rate and mean blood pressure, cardiac arrhythmia, cardiac arrest, apnea, and other typical hemodynamic perturbations, among other autonomic responses^28^. Hence, venous air embolism and TCR should be suspected when rapid bradycardia and hypotension are present with head effects^29^; however, a study of hemodynamic changes after massive pulmonary air embolism in dogs identified no prominent decrease in heart rate and blood pressure or cardiac arrest even after 5 min of model creation^30^. The present results suggest that apnea and the prominent decrease in heart rate and blood pressure were most likely caused by damage to the brainstem, including the medulla oblongata, causing a severe TCR.

Widespread axonal swelling and degeneration characterize traumatic brain injury^31^. Axonal undulations are the first morphological signs of axonal injury, followed by the formation of axonal varicosities^32^. The direct mechanical destruction of microtubules occur at the axonal swelling and axonal varicosity sites, which is associated with a marked decrease in axonal transport. These comprise the typical features of acute axonal injury^33^. Thus, the application of LISW to the upper neck may significantly affect only the region surrounding the medulla oblongata. Further, mice in the Upper neck group experienced respiratory impairment or death, suggesting that pathology was related to impaired axonal transport in the medulla oblongata.

The results of numerous attempts to examine organ-specific responses, as mentioned above, are inconsistent; for example, hypertension occurs in one experimental model and hypotension in another even when the same organ (the brain) is analyzed. This may be explained by the difficulty in specifically applying the shock wave to the chest so as not to affect the head. Thus, in the blast experiment, the application of the shockwaves to a single organ without affecting other organs is technically difficult^34–36^. Two important points should be considered regarding animal studies using rodents to elucidate the mechanisms of traumatic injury attributable to explosions. First, the effects of acceleration attributable to a blast wind, which is known as the tertiary mechanism, should be minimized because many traumatic injuries resulting from explosions, especially Traumatic brain injuries, are known to occur without this mechanism. Thus, it is important to examine the primary mechanism (the effects of the shock wave itself). Second, although actual traumatic injury from explosions is a systemic injury in general, it is often too complex to analyze the outcome of whole-body exposure. Thus, the effects of exposure on the brain and other tissues and organs need to be separated. Because LISW is not accompanied by wind (dynamic pressure) and its energy is spatially confined, both the effects of wind and exposure of other tissues can be excluded in principle. This would offer a unique advantage in using LISWs to study traumatic injury caused by explosions^37^. With shock or blast tubes, it is difficult to completely exclude the tertiary mechanism and exposure of untargeted tissues in animal experiments. The typical durations of IED shock waves have been reported to range from 200 μs to several milliseconds^38^. However, shock waves with such durations have completely different effects on the brains of rodents and humans because of their anatomical differences. The difference in the effects of pressure wave reflection at the brain–skull boundary is crucial. The size of the mouse brain is roughly one-twentieth of that of the human brain^39^. On the basis of the scaling law, we believed that the appropriate shock wave duration to be applied to the mouse brain would be one-twentieth of the aforementioned durations, ranging from 12.5 μs to several hundred microseconds^40^. In this study, experiments were conducted according to the hypothesis that the force product is the most important parameter in determining damage^37^. We are currently studying the possibility of extending the time range, and we need to test the hypothesis with LISW using an optimal time range in the future.

In conclusion, damage to organs such as the brain stem, including the medulla oblongata and others located in the upper dorsal neck, may cause immediate death. Moreover, damage to the brainstem is likely fatal, suggesting that it is critically important to develop equipment that protects the brainstem.

## Data Availability

The data generated and analyzed in this study are available from the corresponding author upon reasonable request.

## References

[1] Tovar MA, Bell RS, Neal CJ (2021). Epidemiology of Blast Neurotrauma: A meta-analysis of blast injury patterns in the military and civilian populations. World Neurosurg..

[2] Clemedson CJ, Hultman HI (1954). Air embolism and the cause of death in blast injuries. Mil. Surg..

[3] Bellamy, R. F. Conventional warfare: Ballistic, blast, and burn injuries. **5**, 295–335 (1991).

[4] Irwin RJ (1999). Shock after blast wave injury is caused by a vagally mediated reflex. J. Trauma.

[5] Miyawaki H (2015). Noradrenalin effectively rescues mice from blast lung injury caused by laser-induced shock waves. Intens. Care Med. Exp..

[6] Sekine Y (2021). Efficacy of body armor in protection against blast injuries using a swine model in a confined space with a blast tube. Ann. Biomed. Eng..

[7] Woischneck D (2009). Respiratory function after lesions in medulla oblongata. Neurol. Res..

[8] Marmarou A (1994). A new model of diffuse brain injury in rats. Part I: Pathophysiology and biomechanics. J. Neurosurg..

[9] Foda MA, Marmarou A (1994). A new model of diffuse brain injury in rats. Part II: Morphological characterization. J. Neurosurg..

[10] Satoh Y (2018). Molecular hydrogen prevents social deficits and depression-like behaviors induced by low-intensity blast in mice. J. Neuropathol. Exp. Neurol..

[11] Kawauchi S, Okuda W, Nawashiro H, Sato S, Nishidate I (2019). Multispectral imaging of cortical vascular and hemodynamic responses to a shock wave: Observation of spreading depolarization and oxygen supply-demand mismatch. J. Biomed. Opt..

[12] Satoh Y (2010). Pulmonary blast injury in mice: A novel model for studying blast injury in the laboratory using laser-induced stress waves. Lasers Surg. Med..

[13] Seno S, Tomura S, Miyazaki H, Sato S, Saitoh D (2021). Effects of selective serotonin reuptake inhibitors on depression-like behavior in a laser-induced shock wave model. Front. Neurol..

[14] Tomura S (2020). A novel mouse model of mild traumatic brain injury using laser-induced shock waves. Neurosci. Lett..

[15] Kimura E (2021). Effect of shock wave power spectrum on the inner ear pathophysiology in blast-induced hearing loss. Sci. Rep..

[16] Ho AM (2002). A simple conceptual model of primary pulmonary blast injury. Med. Hypotheses.

[17] Kuehn R (2011). Rodent model of direct cranial blast injury. J. Neurotrauma.

[18] McIntosh TK (1989). Traumatic brain injury in the rat: Characterization of a lateral fluid-percussion model. Neuroscience.

[19] Cernak I (2004). The pathobiology of moderate diffuse traumatic brain injury as identified using a new experimental model of injury in rats. Neurobiol. Dis..

[20] Ohnishi M, Kirkman E, Guy RJ, Watkins PE (2001). Reflex nature of the cardiorespiratory response to primary thoracic blast injury in the anaesthetised rat. Exp. Physiol..

[21] Wolf SJ, Bebarta VS, Bonnett CJ, Pons PT, Cantrill SV (2009). Blast injuries. Lancet.

[22] Guy RJ, Kirkman E, Watkins PE, Cooper GJ (1998). Physiologic responses to primary blast. J. Trauma.

[23] Cassidy SS (1986). Reflex cardiovascular responses caused by stimulation of pulmonary C-fibers with capsaicin in dogs. J. Appl. Physiol..

[24] Coleridge JC, Coleridge HM (1984). Afferent vagal C fibre innervation of the lungs and airways and its functional significance. Rev. Physiol. Biochem. Pharmacol..

[25] Mishra V (2016). Primary blast causes mild, moderate, severe and lethal TBI with increasing blast overpressures: Experimental rat injury model. Sci. Rep..

[26] Benghanem S (2020). Brainstem dysfunction in critically ill patients. Crit. Care.

[27] Stober T, Sen S, Anstätt T, Bette L (1988). Correlation of cardiac arrhythmias with brainstem compression in patients with intracerebral hemorrhage. Stroke.

[28] Chowdhury T, Petropolis A, Cappellani RB (2015). Cardiac emergencies in neurosurgical patients. Biomed. Res. Int..

[29] Recinos MA, Hsieh J, Mithaiwala H, Mucci JJ, Recinos PF (2021). A rare appearance of the trigeminocardiac reflex during resection of posterior parasagittal meningioma. Surg. Neurol. Int..

[30] Tanus-Santos JE, Gordo WM, Udelsmann A, Cittadino MH, Moreno H (2000). Nonselective endothelin-receptor antagonism attenuates hemodynamic changes after massive pulmonary air embolism in dogs. Chest.

[31] Tang-Schomer MD, Patel AR, Baas PW, Smith DH (2010). Mechanical breaking of microtubules in axons during dynamic stretch injury underlies delayed elasticity, microtubule disassembly, and axon degeneration. Faseb J..

[32] Pernici CD, Kemp BS, Murray TA (2019). Time course images of cellular injury and recovery in murine brain with high-resolution GRIN lens system. Sci. Rep..

[33] Tang-Schomer MD, Johnson VE, Baas PW, Stewart W, Smith DH (2012). Partial interruption of axonal transport due to microtubule breakage accounts for the formation of periodic varicosities after traumatic axonal injury. Exp. Neurol..

[34] Cho SI (2013). Mechanisms of hearing loss after blast injury to the ear. PLoS ONE.

[35] Niwa K (2016). Pathophysiology of the inner ear after blast injury caused by laser-induced shock wave. Sci. Rep..

[36] Svetlov SI (2010). Morphologic and biochemical characterization of brain injury in a model of controlled blast overpressure exposure. J. Trauma.

[37] Miyai K (2021). Axonal damage and behavioral deficits in rats with repetitive exposure of the brain to laser-induced shock waves: Effects of inter-exposure time. Neurosci. Lett..

[38] Courtney MW, Courtney AC (2011). Working toward exposure thresholds for blast-induced traumatic brain injury: Thoracic and acceleration mechanisms. Neuroimage.

[39] Krafft PR (2012). Etiology of stroke and choice of models. Int. J. Stroke.

[40] Jitsu M (2021). Behavioral and histopathological impairments caused by topical exposure of the rat brain to mild-impulse laser-induced shock waves: impulse dependency. Front. Neurol..

